# Physiological and Yield Responses of Peanut (*Arachis hypogaea* L.) Genotypes Under Well-Watered and Water-Stressed Conditions

**DOI:** 10.3390/plants15081243

**Published:** 2026-04-17

**Authors:** Yogesh Dashrath Naik, Alvaro Sanz-Saez, Charles Chen, Phat Dang, N. Ace Pugh, Andrew Young, Yves Emendack, Naveen Puppala

**Affiliations:** 1 Agricultural Science Center, New Mexico State University, Clovis, NM 88101, USA; yogeshn@nmsu.edu; 2 Department of Crop, Soil and Environmental Sciences, Auburn University, Auburn, AL 36849, USA; cyc0002@auburn.edu; 3 National Peanut Research Laboratory, Agricultural Research Service, United States Department of Agriculture, Dawson, GA 39842, USA; phat.dang@usda.gov; 4 Crop Stress Research Laboratory, United States Department of Agriculture, Lubbock, TX 79401, USA; nicholas.pugh@usda.gov (N.A.P.); andrew.young@usda.gov (A.Y.); yves.emendack@usda.gov (Y.E.)

**Keywords:** drought tolerance, water regime, physiological traits, stomatal conductance, yield, peanut

## Abstract

A large proportion of global peanut cultivation occurs in arid and semiarid environments, where water scarcity poses a major limitation to productivity. Climate change further intensifies this challenge by causing irregular rainfall patterns. This study aimed to investigate the physiological and yield responses of peanut genotypes under well-watered and water-stressed conditions. Seven genotypes, five drought-tolerant (C76-16, Line-8, PI 502120, AU-NPL-17 and AU16-28) and two drought-sensitive (Valencia-C and AP-3) were evaluated under two irrigation regimes across consecutive years (2024 and 2025). Seven yield-associated traits (number of pods per plant, pod length, pod width, pod yield per plant, seed weight, hundred-seed weight and pod yield per plot) along with three physiological traits (stomatal conductance, photosynthetic efficiency and leaf temperature) were measured at three growth stages. Drought stress caused a significant reduction in almost all traits, including pod yield per plot (42–44%) and hundred-seed weight (24–38%). Stomatal conductance showed the greatest reduction at all stages, especially during flowering (31–80%) and pod filling (45–74%) stages. Correlation analysis revealed that yield-related traits were negatively correlated with stomatal conductance at pod-filling under water-stress conditions. Genotypes such as PI 502120, AU-NPL-17 and C76-16 maintained higher yields with less reduction under water-stressed conditions. This study also confirmed that Line-8 employs a water-saver strategy, whereas PI 502120 uses a water-spender mechanism to cope with water stress. Additionally, findings showed that the flowering and pod-filling stages are more severely affected physiologically by drought stress, which likely contributed to the observed yield reduction.

## 1. Introduction

Agriculture is the largest consumer of freshwater, accounting for nearly 70% of global withdrawals and up to 95% in developing countries [1]. This heavy demand places tremendous strain on groundwater reserves, as water tables worldwide are declining rapidly due to reduced rainfall and intensive irrigation practices aimed at sustaining crop yields [2]. Among natural disasters, drought remains one of the most severe and costly, threatening global food security and millions of livelihoods [3]. The combined effects of reduced precipitation, rising temperatures, and increased evapotranspiration often trigger agricultural drought, and these events are expected to become more frequent and intense as populations grow and freshwater resources shrink.

Peanut (*Arachis hypogaea* L.), an important legume cultivated in arid and semiarid regions, is particularly vulnerable to water scarcity, with annual yield losses of about 20% due to drought [4]. Drought adversely impacts multiple plant traits and processes, including molecular, biochemical, physiological and morphological characteristics, depending on its severity and duration [5]. Among the key physiological responses to drought, stomatal regulation plays a central role in plant adaptation to water-limited environments [6]. Stomatal adjustment allows plants to cope with fluctuating environments by regulating CO_2_ uptake and water loss [7]. Open stomata facilitate CO_2_ diffusion into the leaf but also result in transpiration driven by the leaf-to-air water vapor gradient. Under soil water deficit, reduced plant water status can have severe effects at both the leaf and whole-plant levels [7]. To mitigate these risks, plants reduce transpirational water loss by decreasing stomatal conductance (*gs*). However, drought-induced stomatal closure also limits CO_2_ uptake, disrupts photosynthetic electron transport, and promotes the accumulation of reactive oxygen species, leading to oxidative damage and reduced photosynthetic efficiency [6,8,9]. These effects intensify at later developmental stages due to both stomatal and biochemical constraints [10].

The importance of drought tolerance in peanut is well recognized for specific production environments in America [4,11,12], Argentina [13], India [14,15], China [16], and Egypt [17]. In response, breeders are actively targeting multiple physiological and morphological mechanisms, including enhanced water-use efficiency, rapid transpiration regulation, deeper root architecture, higher harvest index, increased chlorophyll content, optimized specific leaf area, improved photosystem II (PSII) efficiency, canopy temperature regulation and *gs* [4,9,18,19,20,21]. These traits are expressed through contrasting drought-response strategies; for example, Zhang et al. [4] found that cultivars Line-8 and AU16-28 were water-saver (isohydric) genotypes that restrict transpiration via tight stomatal control. On the other hand, cultivars AU-NPL-17 and PI 502120 were classified as water-spenders (anisohydric), meaning they maintained transpiration and photosynthesis as soil moisture declined [4]. Combining multiple drought tolerance-associated traits into a single variety and delivering it to farmers involves not only the breeding process but also adoption efforts and considerable financial investment, making it a time- and resource-intensive endeavor. The objective of this study was to evaluate water-saving and water-spending peanut genotypes for morpho-physiological traits under well-watered and water-stressed field conditions, and to quantify the behavior of *gs* and photosynthetic efficiency during long-term drought at different plant growth stages for eastern New Mexico and West Texas regions.

## 2. Results

### 2.1. Weather Conditions and Sensor Responses Under Contrasting Water Regimes

**Figure 1:**
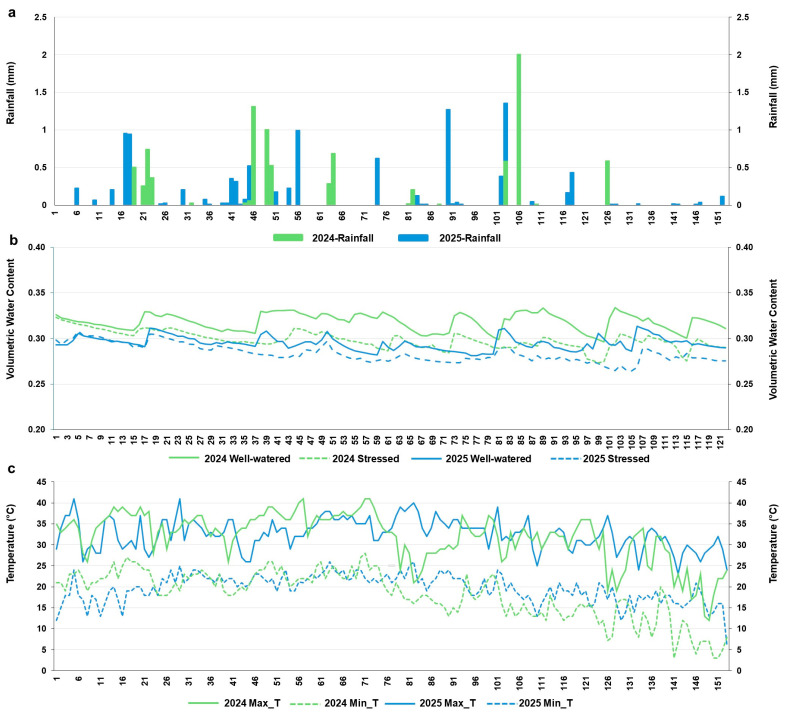
Meteorological observations recorded during the entire crop growth period across two consecutive years (2024 and 2025). (**a**) Rainfall patterns, (**b**) soil moisture content throughout the growing season and (**c**) maximum and minimum temperature variations. The Y-axis represents days after sowing.

Rainfall during the observation period was generally low and occurred in intermittent events in both years (Figure 1a). In 2024, rainfall events were less frequent but included several moderate peaks, whereas in 2025, rainfall was more frequent but with smaller amounts, totaling 358 mm in 2025 and 250 mm in 2024. Volumetric water content values differed clearly between treatments (Figure 1b). The well-watered treatment consistently exhibited higher volumetric water content, typically ranging from 0.31 to 0.34 with periodic fluctuations across the growing period. The persistent separation between the two treatments indicates a sustained difference in sensor response under contrasting water availability conditions in 2025, with greater separation between the well-watered and water-stressed treatments than in 2024. Maximum temperatures remained high throughout the season, ranging from approximately 28 to 40 °C (Figure 1c). In 2025, maximum temperatures showed greater variability and occasional sharp declines later in the season compared to 2024.

### 2.2. Impact of Drought Treatment on Physiological Traits

Field evaluations in 2024 and 2025 showed that drought stress significantly affected the physiological traits of peanut breeding lines across vegetative, flowering and pod-filling stages (Table S1). Leaf temperature increased under stress conditions, while photosynthetic efficiency and *gs* decreased across all stages. Genotype effects were significant for all traits in both years. However, the genotype × treatment interaction was largely non-significant for most traits across years. Physiological traits showed significant treatment effects at flowering and pod-filling stages in both seasons. The magnitude of physiological changes varied with growth stage, with the strongest reductions in *gs* observed during flowering and pod-filling stages. In 2024, average *gs* was reduced under the water-stressed condition at all three growth stages, with the strongest treatment effect observed at the pod-filling stage. Under water stress, *gs* declined by 35.1% at the vegetative stage, 31.6% at flowering, and 45.1% at the pod-filling stage (Table 1). In 2025, the reduction was more pronounced: *gs* declined 45.5% at vegetative, 80.8% at flowering, and 74.2% at the pod-filling stage (Table 2). Drought stress reduced *gs* across all stages, with average reductions of around 37.3% in 2024 and 66.8% in 2025. Photosynthetic efficiency was also affected by water-stressed conditions across growth stages, with reductions varying between years. In 2024, decreases were 20.8% at the vegetative stage, 9.0% at flowering, and 12.6% at the pod-filling stage; in 2025, reductions were 9.7%, 25.9%, and 35.1% at the vegetative, flowering, and pod-filling stages, respectively.

### 2.3. Impact of Drought Treatment on Yield-Related Traits

**Figure 2:**
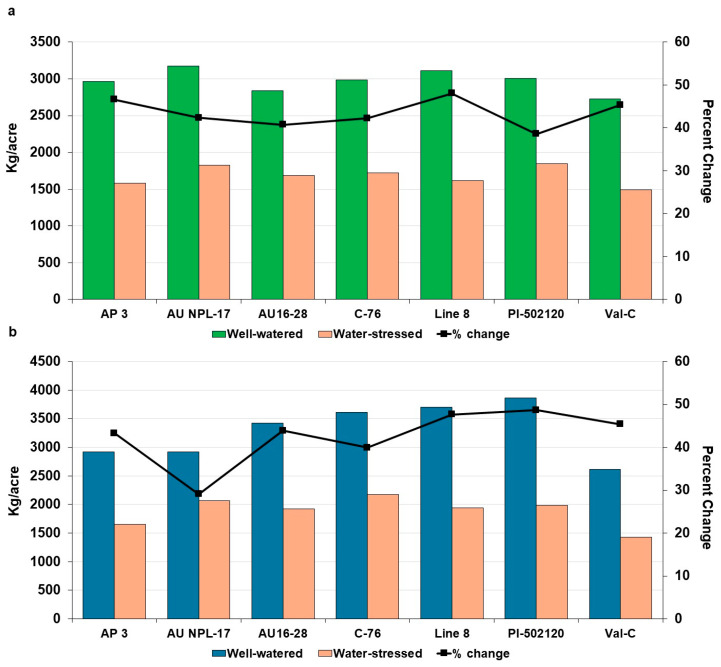
Effects of water stress on pod yield across two growing seasons, with (**a**) representing 2024 and (**b**) representing 2025. The X-axis shows the seven genotypes, and the Y-axis represents pod yield per acre and the percent change under water stress for each genotype.

Drought stress caused significant (*p* < 0.05) reductions in all measured yield-related traits in peanut breeding lines compared with well-watered conditions in both years. In 2024, the number of pods per plant declined sharply under stress (36.7%, Table 1), whereas a comparatively smaller but still significant reduction was observed in 2025 (28.1%). Similar year-dependent trends were evident for pod yield per plant (28.7% in 2024; 36.8% in 2025) and pod yield per plot (43.4% in 2024; 42.9% in 2025). Pod length showed moderate sensitivity, with reductions of 14.7% in 2024 and 13.3% in 2025. Seed-related traits were highly responsive to water stress in both years, with greater reductions in 2024 for seed weight per plant (33.3%) and hundred-seed weight (38.3%), whereas in 2025 the declines were 47.5% and 24.8%, respectively. Genotype-specific impacts on plot yield varied across the two years (Figure 2). In 2024, under well-watered conditions, the highest plot yields were recorded for AU-NPL-17, Line-8, PI 502120, and C76-16, producing 3172, 3114, 3005, and 2984 kg/acre, respectively. Under water-stressed conditions, PI 502120, AU-NPL-17, and C76-16 showed top yields of 1845, 1829, and 1723 kg/acre, with corresponding yield reductions of 38.6%, 42.3%, 42.3%, and 48.1% for PI 502120, AU-NPL-17, C76-16, and Line-8, respectively. In 2025, under well-watered conditions, the highest yields were recorded for PI 502120 (3864 kg/acre), Line-8 (3703 kg/acre), C76-16 (3613 kg/acre), and AU16-28 (3418 kg/acre). Under water-stressed conditions, C76-16 (2170 kg/acre), AU-NPL-17 (2068 kg/acre), and PI 502120 (1983 kg/acre) maintained the top yields. The yield reductions varied among lines, with AU-NPL-17 at 29.1%, C76-16 at 39.9%, AU16-28 at 43.9%, Line-8 at 47.6%, and PI 502120 at 48.7%. Drought-sensitive genotypes Val-C and AP-3 exhibited substantial yield reductions under stress conditions, with Val-C showing declines of 45.3% in 2024 and 45.4% in 2025, and AP-3 showing reductions of 46.6% and 43.3%, respectively. These consistently high losses across both years indicate their susceptibility to drought stress and poor yield stability under water-limited conditions.

### 2.4. Genotype-Specific Performance and Multivariate Analysis Using PCA

**Figure 3:**
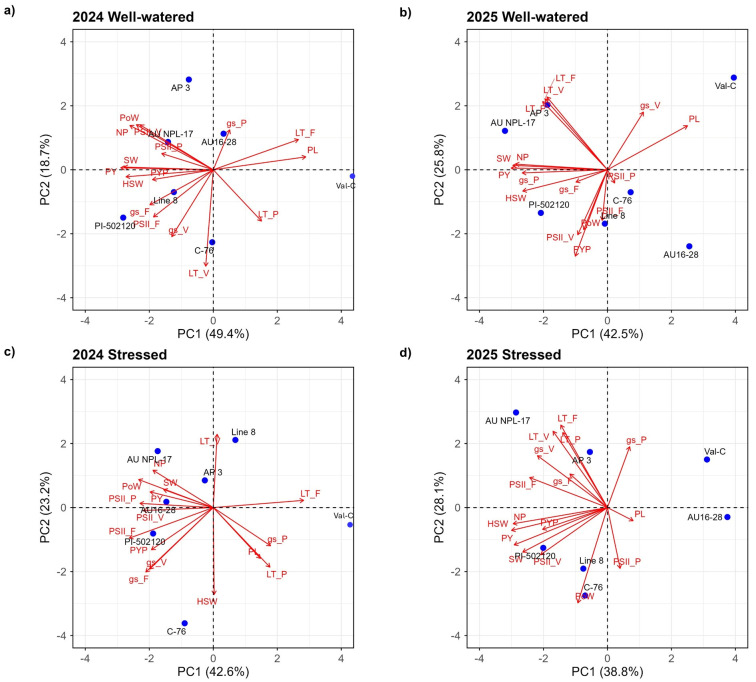
Principal component analysis (PCA) of genotypes under well-watered and stressed conditions in 2024 and 2025. Panels (**a**,**b**) represent the well-watered regime in 2024 and 2025, respectively, and panels (**c**,**d**) represent the water-stressed regime in 2024 and 2025, respectively. Blue dots represent the plotted genotypes. Vectors indicate the direction and magnitude of trait contributions to the principal components. The percentage of total variance explained by each principal component is shown on the respective axes.

The relationships among morpho-physiological traits and genotypes under well-watered and water-stressed conditions in 2024 and 2025 are shown in PCA biplots (Figure 3). The biplots showed clear separation of variables, indicating varied responses among the genotypes. In 2024, PC1 and PC2 explained 49.4% and 18.7% of the total variation under well-watered conditions and 42.6% and 23.2% under stress, respectively. In 2025, PC1 and PC2 accounted for 42.5% and 25.8% (well-watered) and 38.8% and 28.1% (water-stressed), respectively. Under water-stressed conditions in both years, genotypic variation in *gs* and photosystem efficiency was observed at both the flowering and pod-filling stages, with corresponding differences in yield per plot. AU-NPL-17, PI 502120, and C76-16 consistently produced the highest yields across both years.

In the PCA biplot, these genotypes were positioned close to physiological and yield-related traits under both conditions and across years. These genotypes also exhibited higher *gs* during flowering and pod-filling, along with greater photosynthetic efficiency under stress. Among them, AU-NPL-17 showed relatively lower yield reduction of 35.7%. Line-8 consistently exhibited lower *gs* and photosynthetic efficiency under stress across both years, resulting in pronounced yield reduction. Photosynthetic efficiency declined by 27.9% in 2024 and 18.7% in 2025 at flowering. In PCA biplots, Line-8 was positioned opposite *gs* under stress, indicating a negative association. Stomatal conductance sharply declined, with reductions of 79.2% at pod-filling in 2024 and 77.2% in 2025, and 41.7% and 97.7% at flowering in 2024 and 2025, respectively, highlighting its extreme sensitivity to water stress. In contrast, Val-C exhibited the lowest photosynthetic efficiency at both flowering and pod-filling stages, despite relatively high *gs* during pod filling, which was associated with lower yield under stress in both years. Another drought-sensitive genotype, AP-3, was positioned closer to leaf temperature and farther from pod yield per plot in both years under stress, indicating a negative association with yield. Higher leaf temperature may reflect reduced transpirational cooling due to stomatal closure, which can impair photosynthetic activity and ultimately reduce yield. Leaf temperature traits measured across different growth stages clustered closely, with trait vectors positioned close together in both years. Similarly, yield-related traits are grouped, reflecting strong positive correlations and coordinated expression of yield components across genotypes.

### 2.5. Trait Correlations Under Well-Watered and Stressed Conditions

**Figure 4:**
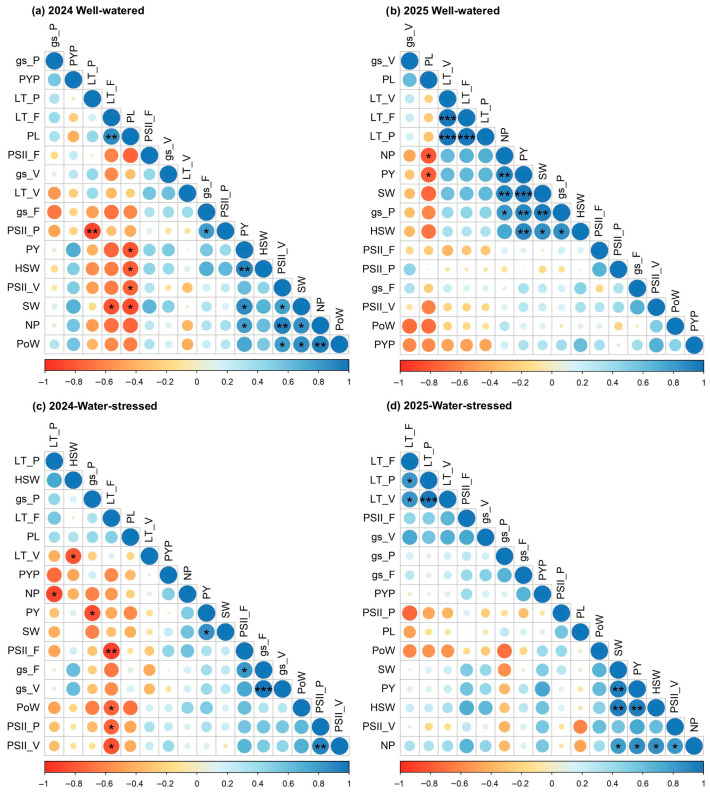
Correlation matrix of evaluated peanut genotypes during the 2024 and 2025 growing seasons under two water regimes. Panels (**a**,**b**) represent the well-watered regime in 2024 and 2025, respectively, while panels (**c**,**d**) represent the water-stressed regime in 2024 and 2025, respectively. Statistical significance is indicated as follows: *** *p* < 0.001, ** *p* < 0.01 and * *p* < 0.05. Stomatal conductance (*gs*), photosystem II efficiency (PSII), and leaf temperature (LT) were measured in vegetative (V), flowers (F) and pod-filling (P) stages. Other traits include number of pods (NP), pod length (PL), pod weight (PoW), pod yield per plant (PY), pod yield per plot (PYP), seed weight (SW) and hundred-seed weight (HSW).

Across both 2024 and 2025, correlation analysis revealed consistent relationships among yield and physiological traits under both water-stressed and well-watered conditions (Figure 4). In 2024, *gs* at flowering showed a significant positive correlation with photosynthetic efficiency at pod-filling. Additionally, photosynthetic efficiency at pod-filling was significantly negatively correlated with leaf temperature at the same stage. Pod yield per plant was positively and significantly correlated with hundred-seed weight, seed weight, and number of pods in both years under well-watered conditions. Under well-watered conditions in both years, pod length showed a significant negative correlation with yield-associated traits, including pod yield per plant and number of pods per plant (Figure 4a,b). Under stressed conditions in 2024, leaf temperature at pod-filling showed a significant negative correlation with the number of pods per plant. Similarly, leaf temperature at flowering exhibited a significant negative association with photosynthetic efficiency across all growth stages. Under stress conditions, *gs* at pod-filling showed a significant negative correlation with pod yield per plant in 2024, whereas in 2025, a negative trend was observed but the association was not statistically significant. Leaf temperature showed a significant negative correlation with yield-related traits, including seed weight per plant, pod yield per plant, number of pods per plant, as well as photosynthetic efficiency (Figure 4c,d).

## 3. Discussion

This study investigated the impact of drought on key physiological traits across different growth stages in peanut genotypes representing contrasting drought-response strategies. Drought tolerance in plants can be achieved either by conserving water through reduced transpiration (water-saver strategy) or by extracting and utilizing more soil water to sustain growth (water-spender strategy) [4,5]. Traits such as high water-use efficiency from rapid stomatal closure and deeper root systems that enhance water uptake play key roles in sustaining canopy carbon assimilation, biomass and yield under drought [5]. The selected genotypes were previously classified as drought-tolerant based on water-saver and water-spender strategies [4]. Under drought conditions, PI 502120 and AU-NPL-17 maintained higher photosynthetic efficiency and *gs* with superior yield, indicating a water-spender strategy, whereas Line-8 and AU16-28 sustained yield with lower stomatal conductance, reflecting a water-saver strategy that conserves water under stress [4]. This contrast enabled a clearer understanding of how drought affects growth, physiology, and yield performance across different developmental stages.

The field evaluation was conducted in western Texas, where the semi-arid climate is characterized by high temperatures and low humidity. These environmental conditions accentuate genotypic differences in drought resilience more than those in the humid southeastern regions, such as Georgia. In the present study, genotypes, treatments, and years all differed significantly for almost all traits, while genotype × treatment interactions were mostly non-significant, indicating stable responses across treatments and facilitating selection in breeding programs. Three growth stages were selected for physiological evaluation, as these are considered the most critical phases influencing peanut performance and yield [22]. This evaluation is more comprehensive and stage-specific under stress conditions, whereas Zhang et al. [4] assessed only a limited set of observations on a few days after drought, at the pod-filling stage. Multiple studies have demonstrated that drought stress significantly reduces both yield and physiological traits from the flowering to pod-filling stages [4,9,12,20,23]. The present study confirmed these findings, showing significant reductions (42–44%) in pod yield per plot under drought stress in both years. These results are consistent with earlier reports documenting reductions of 35.2–57.5% in pod yield under drought conditions [4]. These yield penalties were closely associated with pronounced impairments in physiological traits across all growth stages. The selected physiological traits effectively reflect plant stress responses and genotype performance. These limited traits ensured a manageable scope and reliable measurements. Among these traits, *gs* was the most sensitive parameter and showed a significant reduction at both the flowering and pod-filling stages, with the reduction more pronounced at the flowering stage. These results agree with earlier studies showing that the flowering stage is the most sensitive to drought for *gs* in peanut [10]. Additionally, most physiological traits have been reported to decline under drought conditions [4,6,9]. The severe reduction in *gs* during flowering and pod-filling likely reduced photosynthetic capacity and the supply of assimilates. However, under drought conditions, plants close their stomata to limit water loss and conserve moisture; this conservative strategy enhances plant survival under dry conditions and high evaporative demand [24], and this response was more pronounced in the water-saver genotypes [4].

Correlation analysis revealed that photosynthesis efficiency was positively correlated with *gs* across all conditions. Additionally, a negative relationship was observed between yield-related traits and *gs* at the pod-filling stage under stress conditions. However, earlier studies on peanuts reported a positive correlation with *gs* [4,22]. The differences likely arise from contrasting drought scenarios and environments. Zhang et al. [4] conducted their study in a humid environment, with drought imposed only during pod-filling for about 28 days. Under these conditions, water-spenders could access residual soil moisture through more developed root systems. Higher *gs*, therefore, supported carbon assimilation without major yield loss. In contrast, the present study involved water stress throughout the growing season in an arid environment. Soil moisture was much lower, especially during flowering and reproductive stages. Under these conditions, genotypes with reduced *gs*, particularly at flowering and pod-filling, maintained sustainable yields. This suggests that conservative water-use strategies were beneficial. A similar negative relationship has been reported in *Setaria italica*, where stomatal density was negatively correlated with total above-ground biomass under drought conditions [25]. Reduced *gs* has also been reported to improve water-use efficiency and enhance drought tolerance [26,27].

Stomatal conductance and photosynthetic efficiency differed among genotypes and growth stages under water stress. In 2024, Line-8 exhibited lower *gs*, while PI 502120 displayed higher *gs*. In 2025, Line-8 and AU16-28 showed comparatively lower *gs* with higher yield, whereas PI 502120 and AU-NPL-17 maintained relatively higher *gs*. These results are consistent with Zhang et al. [4] and indicate that Line-8 is a water-saver, while PI 502120 is a water-spender. AU16-28 and AU-NPL-17 exhibited contrasting responses in both years. The drought-sensitive genotypes, AP-3 and Valencia-C, exhibited pronounced reductions in most physiological and yield-related traits under water stress. AP-3 showed severe declines in *gs* during flowering (41–97%) and pod-filling (34–84%) across 2024 and 2025, accompanied by substantial reductions in pod yield per plot (46–44%) and hundred-seed weight (26–40%). Similarly, Valencia-C displayed marked decreases in *gs*, particularly during vegetative growth (65–67%) and flowering (48–79%), along with notable yield losses (45–46%). Both genotypes also experienced reductions in photosynthetic efficiency and leaf temperature regulation, indicating impaired physiological performance under drought. These results indicate that AP-3 and Valencia-C are more sensitive to water deficit, highlighting their limited capacity to maintain gas exchange and yield under stress conditions.

When data from all growth stages were combined for each year, considerable variation in *gs* was observed among genotypes. AU-NPL-17 maintained moderate *gs* and high yield, reflecting efficient water use. PI 502120 had slightly higher *gs* with moderate yield, indicating a balanced strategy. Line-8 exhibited the lowest gs, showing strong water-saving behavior, while AU16-28 had intermediate *gs* and moderate yield, demonstrating flexible water-use under varying conditions. Low *gs* under drought stress contributes to maintaining yield by minimizing transpirational water loss while optimizing water-use efficiency [4]. Reduced *gs* limits the diffusion of water vapor from the leaf to the atmosphere, conserving soil moisture for critical growth and reproductive stages. At the same time, moderate *gs* allows sufficient CO_2_ uptake to sustain photosynthetic carbon assimilation and maintain PSII efficiency, thereby preserving source strength and partitioning of assimilates to reproductive organs [28]. The ability to sustain photosynthetic efficiency under water deficit appears to be a key drought-tolerance mechanism in peanuts, contributing to higher simulated rainfed yields and making these genotypes valuable for dryland cultivation [4]. An increase in leaf temperature was also observed under drought stress, likely due to stomatal closure, which reduces transpiration and latent heat dissipation, thereby increasing leaf temperature [21]. Future studies could expand both the number of genotypes and the range of measured traits, including enzyme activities and other physiological or biochemical indicators, to achieve a more comprehensive understanding of stress responses.

## 4. Materials and Methods

### 4.1. Plant Material and Growth Conditions

The experiment was conducted as a randomized complete block design (RCBD) with four replications at the USDA station in Lubbock, Texas, USA (33.585° N, 101.905° W). A total of seven advanced breeding lines were used in this study (Table 3). Each plot measured about 4.0 m in length and 1.0 m in width and consisted of two side-by-side rows. The experiment was conducted over two consecutive years (2024 and 2025). Peanut genotypes were sown on 2 May 2024 and 23 April 2025 and evaluated under two contrasting water treatments (well-watered and water-stressed). The soil sensor array was 0.8 m long, with 8 sensors spaced 0.10 m apart [29,30]. The data were remotely accessed using a GoField^®^ Irrigation Scheduling Solution device (Goanna Ag, Goondiwindi, Australia). For data analysis, only the readings from five upper sensors were used to calculate soil moisture, as these corresponded to the depth range occupied by peanut roots. Plants under the well-watered treatment received four supplemental irrigations during the growing season, scheduled when the average soil matric potential reached −60 kPa. Each event delivered 25.5 mm of water, while under the water-stressed condition, irrigation was restricted to 12.5 mm per event.

### 4.2. Leaf Gas Exchange and Chlorophyll Fluorescence Measurements

Stomatal conductance (*gs*) was measured on adaxial leaf surfaces using an LI-600 Porometer (LI-COR Biosciences, Lincoln, NE, USA). Simultaneous measurements of the effective quantum yield of photosystem II (ΦPSII/photosynthetic efficiency) and leaf temperature (LT) were also obtained using the same instrument. Measurements were taken in both years at 40, 60 and 90 days after sowing, corresponding to the vegetative, flowering and pod-formation stages, respectively. Two measurements per plot were performed in different plants and then averaged to obtain the plot mean used for the statistical analysis. To minimize environmental variation, data were collected between 09:30 and 11:30 a.m. using the youngest fully expanded, sun-exposed leaves from the upper canopy to reduce the influence of leaf age and position. All measurements were performed using the default instrument settings with the auto-stabilization feature enabled. The flow rate was set to “high” (150 µmol s^−1^), the flash type to “rectangular” with an intensity of 7000 µmol m^−2^ s^−1^, and the fluorescence constants “Leaf absorptance” and “Fraction Abs PSII” were fixed at 0.8 and 0.5, respectively. The actinic modulation rate was maintained at 500 Hz.

### 4.3. Morphological Characterization of Peanut Genotypes

At harvest, three plants were randomly selected from each plot for recording morphological and yield-related traits. Yield-related traits included the number of pods per plant (NP), pod length (PL), pod width (PoW), pod yield per plant (YP), seed weight (SW) and hundred-seed weight (HSW). The total number of pods was counted manually for each plant. Moreover, fully developed, three representative pods were selected, and their dimensions (PL and PoW) were measured using a digital vernier caliper (OMETOOLS, Hangzhou, China). The measurements were then averaged to obtain the plot mean used for statistical analysis. The remaining plants in each plot were mechanically harvested 150 days after sowing, collected separately, and used to calculate pod yield per plot (PYP).

### 4.4. Statistical Analysis

Data collected across all treatments were analyzed using a three-way analysis of variance (ANOVA), with year, treatments and genotypes as main factors. Each treatment included four replications per trait, and the ANOVA was performed using GENSTAT 15th Edition (Rothamsted Experimental Station; [34]). Correlation analysis was performed using IBM SPSS Statistics (version 30, IBM Corp., Armonk, NY, USA). The “ggplot2” (version 4.0.2) and “corrr” (version 0.4.5) R packages (version 4.6.0) were used to prepare boxplots and correlation plots. Principal component analysis (PCA) was performed using the R packages “FactoMineR” (version 2.14) and “factoextra” (version 2.0) to visualize the multivariate variation among peanut genotypes under well-watered and water-stressed conditions.

## 5. Conclusions

This study evaluated the physiological responses of seven peanut breeding lines to drought stress under field conditions. The study suggests that drought stress significantly impacts most traits, including pod yield per plot, seed yield, and hundred-seed weight. Physiological traits, particularly *gs*, were the most affected, showing pronounced reductions during flowering and pod-filling, indicating that these developmental stages are especially sensitive to water stress. Best-performing genotypes (e.g., AU-NPL-17, C76-16, and PI-502120) maintained higher *gs*, PSII efficiency, and yield components, whereas sensitive genotypes (AP-3 and Val-C) exhibited reduced physiological performance and substantially lower pod and seed yield under stress compared to well-watered conditions. The study also indicates that Line-8 behaves as a water-saver, whereas PI 502120 shows water-spender-like behavior. Additionally, tolerant genotypes mitigate drought risks by reducing transpirational water loss through decreased *gs*, thereby enhancing survival and maintaining yield. This study highlights the potential to enhance peanut drought tolerance by regulating *gs* and photosynthetic efficiency, offering a pathway for the development of drought-tolerant peanut cultivars.

## Figures and Tables

**Table 1:** Descriptive statistics and significance summary for genotype (G), water treatment (T), and their interaction (G × T) for various growth, yield, and physiological traits in peanut under well-watered and water-stressed conditions in 2024.

Traits	Well-Watered			Water-Stressed			% Change	*p* Value (Fisher’s Test)		
	Mean ± SEM	Min	Max	Mean ± SEM	Min	Max		G	T	G × T
Number of pods per plant	41.5 ± 2.41	24.5	49.5	26.3 ± 1.75	17.3	33.9	36.7	***	***	ns
Pod length (cm)	3.4 ± 0.06	3.1	3.9	2.9 ± 0.06	2.6	3.1	14.7	***	***	ns
Pod width (cm)	1.4 ± 0.03	1.2	1.5	1.3 ± 0.02	1.2	1.4	4.8	**	*	ns
Pod yield per plant (g)	43.2 ± 2.63	22.8	54.3	30.8 ± 1.67	18.4	39.4	28.7	***	***	ns
Seed weight per plant (g)	36.8 ± 2.32	16.5	48.0	24.5 ± 1.8	12.6	36.3	33.3	***	***	*
Hundred-seed weight (g)	46.1 ± 1.68	32.0	51.6	28.5 ± 1.43	23.4	39.5	38.3	***	***	**
Pod yield per plot (kg/acre)	2972 ± 87	2727	3172	1682 ± 81	1490	1845	43.4	*	***	ns
Leaf temperature (LT) ^a^	28.3 ± 0.52	26.2	30.0	31.6 ± 0.53	28.3	33.3	−11.7	**	***	ns
Photosynthetic efficiency (PSII) ^a^	0.6 ± 0.01	0.6	0.7	0.5 ± 0.03	0.6	0.6	20.8	***	***	ns
Stomatal conductance (*gs*) ^a^	0.1 ± 0.02	0.1	0.3	0.1 ± 0.01	0.1	0.2	35.1	***	***	ns
Leaf temperature (LT) ^b^	27.8 ± 0.50	25.6	30.0	29.4 ± 0.65	27.8	32.5	−6.0	**	***	ns
Photosynthetic efficiency (PSII)	0.6 ± 0.02	0.6	0.7	0.6 ± 0.02	0.5	0.6	9.0	***	***	ns
Stomatal conductance (*gs*) ^b^	0.2 ± 0.02	0.1	0.3	0.1 ± 0.02	0.1	0.2	31.6	***	***	ns
Leaf temperature (LT) ^c^	26.9 ± 0.46	24.6	28.6	26.9 ± 0.57	26.9	30.9	−7.7	**	***	ns
Photosynthetic efficiency (PSII) ^c^	0.5 ± 0.03	0.4	0.6	0.5 ± 0.03	0.3	0.6	12.6	***	***	ns
Stomatal conductance (*gs*) ^c^	0.1 ± 0.01	0.0	0.1	0.13 ± 0.01	0.03	0.12	45.1	***	***	ns

Note: The significance of genotype (G), treatment (T), and their interaction (G × T) was determined using analysis of variance (ANOVA). Statistical significance is indicated as follows: *** *p* < 0.001, ** *p* < 0.01, * *p* < 0.05 and ns (not significant). ^a^ Vegetative stage, ^b^ flowering stage and ^c^ pod-filling stage.

**Table 2:** Descriptive statistics and significance summary for genotype (G), water treatment (T), and their interaction (G × T) for various growth, yield, and physiological traits in peanut under well-watered and water-stressed conditions in 2025.

Traits	Well-Watered			Water-Stressed			% Change	*p* Value (Fisher’s Test)		
	Mean ± SEM	Min	Max	Mean ± SEM	Min	Max		G	T	G × T
Number of pods per plant	37.8 ± 2.54	24.4	52.5	27.1 ± 1.63	18.8	32.8	28.1	**	***	ns
Pod length (cm)	3.6 ± 0.07	3.2	4.2	3.1 ± 0.06	2.8	3.5	13.3	***	***	ns
Pod width (cm)	1.5 ± 0.03	1.4	1.6	1.4 ± 0.02	1.3	1.5	9.6	*	**	ns
Pod yield per plant (g)	50.6 ± 4.27	28.3	68.7	32.0 ± 2.29	22.5	38.7	36.8	**	***	ns
Seed weight per plant (g)	31.4 ± 2.78	16.5	45.9	16.5 ± 0.94	12.0	19.8	47.5	**	***	ns
Hundred-seed weight (g)	54.6 ± 2.13	43.9	64.6	41.0 ± 2.38	26.5	51.3	24.8	**	***	ns
Pod yield per plot (kg/acre)	3293 ± 144	2671	3864	1880.7 ± 75	1430	2170	42.9	**	***	*
Leaf temperature (LT) ^a^	27.2 ± 0.83	24.1	30.4	27.3 ± 0.90	24.2	30.7	−0.5	***	ns	ns
Photosynthetic efficiency (PSII) ^a^	0.5 ± 0.03	0.4	0.7	0.5 ± 0.02	0.4	0.5	9.7	*	*	ns
Stomatal conductance (*gs*) ^a^	0.3 ± 0.04	0.2	0.5	0.2 ± 0.03	0.1	0.3	45.5	*	***	ns
Leaf temperature (LT) ^b^	27.1 ± 0.82	24.4	30.1	28.1 ± 0.77	25.2	31.1	−3.4	***	ns	ns
Photosynthetic efficiency (PSII) ^b^	0.5 ± 0.02	0.4	0.6	0.3 ± 0.02	0.2	0.4	25.9	*	*	ns
Stomatal conductance (*gs*) ^b^	0.2 ± 0.04	0.1	0.4	0.1 ± 0.01	0.0	0.1	80.8	*	***	ns
Leaf temperature (LT) ^c^	27.2 ± 0.88	24.4	30.6	27.6 ± 1.09	24.6	32.6	−1.4	***	ns	ns
Photosynthetic efficiency (PSII) ^c^	0.5 ± 0.01	0.5	0.6	0.3 ± 0.04	0.2	0.5	35.1	*	*	ns
Stomatal conductance (*gs*) ^c^	0.2 ± 0.04	0.1	0.4	0.1 ± 0.01	0.0	0.1	74.2	*	***	ns

Note: The significance of genotype (G), treatment (T), and their interaction (G × T) was determined using analysis of variance (ANOVA). Statistical significance is indicated as follows: *** *p* < 0.001, ** *p* < 0.01, * *p* < 0.05 and ns (not significant). ^a^ Vegetative stage, ^b^ flowering stage and ^c^ pod-filling stage.

**Table 3:** Peanut genotypes used in the study, their pedigree, water-use strategy (water saver or spender) and drought response.

Genotype	Pedigree	Water Saver/Spender	Drought Response	Origin	References
AP-3	OKFH15 × NC3033	N/A	S	USA	[31,32]
AU16-28	C76-16 × AT-3085-RO	Saver	T	USA	[4]
AU-NPL-17	Tifguard × York	Spender	T	USA	[4]
C76-16	Germplasm selection	N/A	T	USA	[4]
Line-8	C76-16 × Georgia Green	Saver	T	USA	[4]
PI 502120	Landrace	Spender	T	Peru	[4]
Valencia-C	Irradiated population of Colorado Manfredi	N/A	S	USA	[33]

Note: Susceptible (S); Tolerant (T); Not classified as water saver and water spender (N/A).

## Data Availability

The data that support the findings of this study are available from the corresponding authors upon reasonable request.

## Supplementary Materials

The following supporting information can be downloaded at: https://www.mdpi.com/article/10.3390/plants15081243/s1, Table S1: Mean values of physiological and agronomic traits of peanut genotypes under well-watered and drought-stress conditions across two years (2024–2025).

## Abbreviations

*gs*, Stomatal conductance; HSW, Hundred seed weight; LT, Leaf temperature; NP, Number of pods per plant; PL, Pod length; PSII, Photosynthetic efficiency; PoW, Pod width; PYP, Pod yield per plant; PYP, Pod yield per plot; SW, Seed weight per plant.
